# Rb/Sr isotopic and compositional retentivity of muscovite during deformation

**DOI:** 10.1016/j.lithos.2015.04.007

**Published:** 2015-06-15

**Authors:** T. Eberlei, G. Habler, W. Wegner, R. Schuster, W. Körner, M. Thöni, R. Abart

**Affiliations:** a Department of Lithospheric Research, University of Vienna, Althanstraße 14, A-1090 Vienna, Austria; b Austrian Geological Survey, Neulinggasse 38, A-1030 Vienna, Austria; c Department of Environmental Geosciences, University of Vienna, Althanstraße 14, A-1090 Vienna, Austria

**Keywords:** Rb–Sr geochronology, Permian metapegmatites, Upper-greenschist facies deformation, Magmatic muscovite

## Abstract

Permian metapegmatite muscovite from the Upper-Austroalpine Matsch Unit in Southern Tyrol (Italy) was investigated regarding its Rb/Sr and compositional retentivity during Cretaceous Upper-greenschist facies deformation. The data imply that microstructurally relic Permian magmatic muscovite largely maintained its major and trace element compositions during deformation, whereas the Rb/Sr geochronometer is strongly affected by a net loss of Sr. Lower Sr concentrations of muscovite correlate with higher ^87^Rb/^86^Sr and ^87^Sr/^86^Sr ratios. In most samples, the muscovite grain size- and magnetic-fractions with the lowest ^87^Rb/^86^Sr and ^87^Sr/^86^Sr ratios preserve a Permo-Triassic muscovite–whole rock Rb/Sr apparent age interpreted as to reflect formation during or cooling after pegmatite emplacement. Contrastingly, muscovite fractions with higher ^87^Rb/^86^Sr and ^87^Sr/^86^Sr ratios are arranged along a roughly linear array with a positive correlation of the ^87^Rb/^86^Sr and ^87^Sr/^86^Sr ratios in the ^87^Rb/^86^Sr vs ^87^Sr/^86^Sr space. They yield successively lower muscovite–whole rock Rb/Sr apparent ages. We explain the variations in the Rb/Sr isotopic character of microstructurally relic muscovite by a, presumably deformation-related, loss of Sr during the Cretaceous event. Contemporaneously, only very limited amounts of isotopically different Sr from the matrix reservoir might possibly have entered the muscovite. Consequently, the Rb/Sr of the relic muscovite is affected by a net loss of Sr. The results imply that at temperatures of < 500 °C, deformation is supposed to be the predominant factor in controlling the Rb/Sr geochronometer of relic muscovite, by significantly reducing the characteristic length scale for volume diffusion. However, variations of the major and trace element compositions within Permian relic muscovite are interpreted to rather reflect primary compositional instead of deformation-related variations.

## Highlights

• At < 500 °C, deformation is supposed to be the predominant factor in controlling the Rb/Sr geochronometer of relic muscovite
• The Rb/Sr geochronometer of deformed relic muscovite is significantly affected by a net loss of Sr
• The major and trace element composition of relic muscovite is supposed to reflect primary compositional variations
• Following the Permian–Triassic event, the pegmatites cooled below 500 °C at 260–240 Ma

## 1 Introduction

As one of the most common minerals in the Earth's crust, muscovite is stable in various igneous and metamorphic rock types and a wide range of P–T conditions. The Rb–Sr geochronometer in muscovite has commonly been used to infer cooling, (neo-)crystallisation and recrystallisation ages, often in conjunction with the associated whole rock or other coexisting phases (e.g., Armstrong et al., 1966, Bröcker et al., 2013, Freeman et al., 1997, George and Bartlett, 1996, Glodny et al., 2003, Glodny et al., 2002, Glodny et al., 1998, Glodny et al., 2008, Jäger, 1967, Kühn et al., 2000, Müller et al., 1999, Müller et al., 2000, Thöni, 1981). The original concept of the ‘blocking’ or ‘closure’ temperature (Dodson, 1973, Jäger, 1967) implies that temperature is the rate-limiting factor for isotope mobility and that isotopes leaving the crystal by temperature-dependent volume diffusion are exchanged with an infinite reservoir. Other important factors are the effective diffusion domain size (i.e., grain size), dynamic recrystallisation, the cooling rate, the diffusion coefficients for elements and isotopes in the mineral under investigation, the modal composition of a rock, the presence or absence of grain boundary fluids which could influence rates of material exchange with an infinite reservoir, chemical exchange among different minerals during cooling and the mineral composition (Ganguly and Ruiz, 1987, Glodny et al., 2003, Jenkin, 1997, Jenkin et al., 2001, Jenkin et al., 1995, Villa, 1998, Yund and Tullis, 1991). For example, Kühn et al. (2000) found that biotite largely preserved its Neoproterozoic Rb–Sr ages in rocks which had experienced eclogite facies conditions in the Ordovician at temperatures exceeding 650 °C, which they attributed to the absence of a free fluid phase during metamorphism. The Rb–Sr isotopic system of cm-sized, undeformed muscovite from granitic metapegmatites in the western Bohemian massif remained closed at temperatures exceeding 600 °C (Glodny et al., 1998). Commonly reported closure temperatures for the Rb–Sr geochronometer in white micas are in the range of 500 °C to > 600 °C (e.g., Blanckenburg et al., 1989, Freeman et al., 1997, Glodny et al., 2008, Purdy and Jäger, 1976). Glodny et al. (1998) also identified plastic deformation at these temperatures in shear zones as cause for resetting the Rb–Sr isotopic system of muscovites. In ^40^Ar–^39^Ar geochronology, it is well known, that dislocations, subgrain boundaries, kinks and stacking faults can act as fast-diffusion pathways and therefore influence the Ar-retentivity of crystals at temperatures below the closure temperature of Ar diffusion for pristine crystals of a given size (e.g., Baldwin and Lister, 1998, Cosca et al., 2011, Dunlap and Kronenberg, 2001, Hames and Cheney, 1997, Kramar et al., 2003, Mulch et al., 2002, Reddy et al., 1996). It is also known, that cryptic recrystallisation during metamorphism can influence the Ar-retentivity (Beltrando et al., 2013).

Permian metapegmatites in the Austroalpine Matsch Unit (Ötztal–Stubai Basement Complex, OSC) in Southern Tyrol (Italy) were overprinted by localised shear deformation at upper-greenschist facies conditions of c. 500 °C and 5 kbar during the Cretaceous Eo-Alpine tectonometamorphic event (Habler et al., 2009, Schmid and Haas, 1989). Therefore, the Permian metapegmatites provide excellent natural examples to study the Rb–Sr isotopic and compositional retentivity of coarse-grained microstructurally relic muscovite clasts and the mechanisms affecting their Rb–Sr system during deformation. For this purpose, we used different bulk mineral separates from several hand specimens (6 in total). Different muscovite grain-size and magnetic-fractions were used for the combined analysis of Rb and Sr by ID-TIMS and the major and trace elements by EPMA, ICP-OES and ICP-MS. The new data provide insights into the behaviour of the Rb–Sr system and major and trace elements in Permian muscovite porphyroclasts during Cretaceous deformation.

## 2 Geological setting

**Fig. 1:**
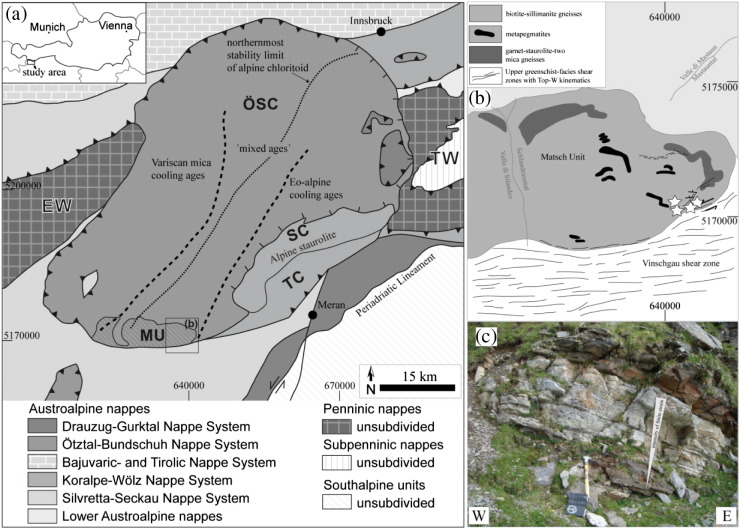
(a) Simplified geological sketch map of the Upper-Austroalpine Ötztal–Stubai Crystalline complex based on Schmid et al., (2004) and Thöni (1981). Abbreviations: EW = Engadine window; TW = Tauern window; OSC = Ötztal–Stubai Complex; SC = Schneeberg Complex; TC = Texel Complex; MU = Matsch Unit. (b) Simplified sketch map of the eastern portion of the Matsch Unit (based on Habler et al., 2009) with white stars marking the locations of the four sampled meta-pegmatite bodies (cf. Table 1). (c) Representative metapegmatite outcrop within the biotite–sillimanite gneisses. Coordinates are UTM Zone 32T, WGS 84.

Samples of Permian metapegmatites were collected in the Matsch Unit in Southern Tyrol (Italy). A list of studied samples with UTM coordinates (UTM Zone 32T, WGS84) is given in Table 1. The Matsch Unit is located at the southern margin of the Upper-Austroalpine Ötztal–Stubai Crystalline complex (OSC, Fig. 1a). The km-wide ‘Vinschgau Shear Zone’ (Schmid & Haas, 1989) defines its southern tectonic boundary. Eo-Alpine T-conditions are supposed to gradually increase from W to E in shear direction of the Vinschgau Shear Zone. The predominant lithologies in the Matsch Unit are biotite–sillimanite gneisses and garnet–staurolite–two mica schists with frequently intercalated Permian metapegmatites (Fig. 1b–c, Ratschiller, 1953). Cretaceous deformation is localised in shear zones and characterised by north-dipping foliations, E–W trending fold axes and stretching lineations of quartz and feldspar, locally varying gradients of finite-strain at the cm- to m-scale and top-W shear kinematics (Schmid & Haas, 1989). The polymetamorphic evolution of the Matsch Unit is characterised by Carboniferous amphibolite facies regional metamorphism, a Permian HT/LP event related with pegmatite formation and finally upper-greenschist facies tectonometamorphism culminating in P–T conditions of 480 ± 26 °C at 4 ± 1.6 kbar (Habler et al., 2009). The timing of pegmatite emplacement was constrained to 263–280 Ma by Sm–Nd garnet–whole rock data (Habler et al., 2009). Eo-Alpine deformation was dated at 83 ± 1 Ma based on Rb–Sr thin slab data of mylonitic meta-pegmatites (Thöni, 1986). Eo-Alpine metamorphism in the OSC formed a sequence of NE–SW trending mineral zones which document increasing Cretaceous metamorphism from NW to SE by: (i) a zone without Cretaceous metamorphic mineral content, (ii) a Stilpnomelane zone, (iii) a Chloritoid zone (Purtscheller, 1967) and (iv) the Eo-Alpine Staurolite zone (Thöni, 1981, Thöni, 1983). These zones correlate with characteristic K–Ar and Rb–Sr biotite ages (Thöni, 1981). Zone (i) correlates with Carboniferous biotite cooling ages (Fig. 1a). The stilpnomelane and chloritoid zones are characterised by opening and incomplete resetting of the K–Ar isotopic system in biotite, or excess ^40^Ar in biotite (Fig. 1a, Thöni, 1981) whereas the zone of Eo-Alpine staurolite correlates with Late Cretaceous biotite cooling ages. Regarding the regional distribution of Cretaceous minerals, the studied rocks of the Matsch Unit are part of the chloritoid zone, wherefrom biotite K–Ar isotopic data showed disturbance but no complete Cretaceous isotopic resetting (Thöni, 1981, Thöni, 1983).

**Table 1:** Sampling locations (UTM Zone 32T, WGS 84) of the studied samples. Sampling locations are given with an accuracy of ± 10 m. They are also shown with white stars in Fig. 1a. Note that HM00305 and the three samples M1203, M1201 and M1206 are from different outcrops of the same pegmatite body (cf. Fig. 1c).

Sample	Rock type	North [m]	East [m]	Altitude [m]
M1210	Meta-pegmatite	5,170,589	640,300	2830
M1217	Meta-pegmatite	5,170,206	640,396	2680
HM00305	Protomylonitic meta-pegmatite	5,170,494	641,263	2596
M1203	Protomylonitic meta-pegmatite	5,170,319	640,984	2624
M1201	Mylonitic meta-pegmatite	5,170,319	640,984	2624
M1206	Ultramylonitic meta-pegmatite	5,170,310	640,963	2620

The metapegmatites are commonly intercalated in biotite–sillimanite gneisses, rarely in Grt–St micaschist (Fig. 1b–c). The primary magmatic mineral assemblage of the pegmatites consists of quartz + albite + muscovite ± garnet ± K-feldspar ± apatite ± accessory zircon and monazite. The metamorphic mineral assemblage contains albite + quartz + muscovite ± K-feldspar ± apatite ± biotite ± garnet ± clinozoisite/allanite. Despite intense deformation, muscovite and feldspar clasts of the primary magmatic mineral assemblage were preserved as microstructural relics.

## 3 Analytical methods

### 3.1 Electron microprobe (EPMA)

Compositional mineral analyses were performed at the Department of Lithospheric Research at the University of Vienna using a Cameca SX100 instrument with an acceleration voltage of 15 keV, a beam current of 20 nA and a beam diameter of 3 μm for white mica and apatite and 6 μm for feldspar. Natural and synthetic standards were used for calibration. The PAP routine (Pouchou & Pichoir, 1991) was used for matrix corrections. Mineral formulae of white mica are normalised to 11 oxygen and assuming all Fe as Fe^2 +^. Element distribution maps were obtained by continuous stage movement with dwell times of 40 ms and a step size of 1 μm. Representative muscovite analyses are given in Table 2.

**Table 2:** Representative EPMA analyses of muscovite.

Wm group	I	II	III	I	II	III	I	II	III	I	II	III	I	II	III
Generation	Permian	Cretaceous	Cretaceous	Permian	Cretaceous	Cretaceous	Permian	Cretaceous	Cretaceous	Permian	Cretaceous	Cretaceous	Permian	Cretaceous	Cretaceous
Sample		M1217			HM00305			M1203			M1201			M1206	
Position	Clast	Clast rim	Matrix	Clast	Clast rim	Matrix	Clast	Clast rim	Matrix	Clast	Clast rim	Matrix	Clast	Clast rim	Matrix
SiO_2_	45.93	47.24	46.38	45.62	46.71	48.18	45.52	46.76	47.36	45.87	47.00	46.13	47.01	47.25	46.76
TiO_2_	0.09	0.08	0.08	0.05	0.04	0.02	0.08	0.18	0.08	0.01	0.11	0.10	1.35	0.43	0.01
Al_2_O_3_	35.68	32.43	34.47	36.25	32.29	32.60	35.37	31.13	30.98	36.95	34.35	35.14	34.52	31.75	32.67
Cr_2_O_3_	0.01	0.00	0.00	0.00	0.00	0.00	0.00	0.00	0.01	0.00	0.00	0.00	0.00	0.00	0.00
FeO	2.48	3.52	2.60	2.16	3.92	4.23	2.17	4.94	4.44	1.29	2.71	2.51	3.44	4.23	4.25
MnO	0.00	0.02	0.01	0.00	0.03	0.04	0.01	0.04	0.02	0.02	0.03	0.01	0.01	0.00	0.02
MgO	0.42	1.01	0.67	0.30	0.67	1.02	0.35	1.07	1.12	0.38	0.75	0.50	0.47	0.65	0.48
CaO	0.01	0.08	0.03	0.01	0.00	0.00	0.00	0.00	0.00	0.00	0.00	0.01	0.00	0.00	0.00
Na_2_O	0.25	0.17	0.34	0.40	0.20	0.17	0.38	0.25	0.12	0.59	0.49	0.53	0.35	0.35	0.45
K_2_O	10.83	10.48	10.73	10.69	10.56	10.19	10.80	10.77	11.10	10.70	10.38	10.26	10.64	10.57	10.23
Total	95.69	95.02	95.30	95.48	94.42	96.46	94.69	95.14	95.22	95.81	95.81	95.18	97.79	95.24	94.88
Oxygens	11.00	11.00	11.00	11.00	11.00	11.00	11.00	11.00	11.00	11.00	11.00	11.00	11.00	11.00	11.00
Si	3.06	3.18	3.11	3.04	3.18	3.20	3.07	3.18	3.21	3.04	3.13	3.09	3.08	3.19	3.16
Ti	0.00	0.00	0.00	0.00	0.00	0.00	0.00	0.01	0.00	0.00	0.01	0.00	0.07	0.02	0.00
Al	2.80	2.57	2.72	2.85	2.59	2.55	2.81	2.50	2.48	2.88	2.69	2.77	2.67	2.53	2.61
Cr	0.00	0.00	0.00	0.00	0.00	0.00	0.00	0.00	0.00	0.00	0.00	0.00	0.00	0.00	0.00
Fe-II	0.14	0.20	0.15	0.12	0.22	0.23	0.12	0.28	0.25	0.07	0.15	0.14	0.19	0.24	0.24
Mn	0.00	0.00	0.00	0.00	0.00	0.00	0.00	0.00	0.00	0.00	0.00	0.00	0.00	0.00	0.00
Mg	0.04	0.10	0.07	0.03	0.07	0.10	0.04	0.11	0.11	0.04	0.07	0.05	0.05	0.07	0.05
Ca	0.00	0.01	0.00	0.00	0.00	0.00	0.00	0.00	0.00	0.00	0.00	0.00	0.00	0.00	0.00
Na	0.03	0.02	0.04	0.05	0.03	0.02	0.05	0.03	0.02	0.08	0.06	0.07	0.04	0.05	0.06
K	0.92	0.90	0.92	0.91	0.92	0.86	0.93	0.93	0.96	0.90	0.88	0.88	0.89	0.91	0.88
Total	7.01	6.99	7.01	7.01	7.00	6.97	7.01	7.05	7.03	7.01	6.99	7.00	6.99	7.00	7.00

### 3.2 Rb/Sr isotope dilution analysis (ID-TIMS)

Selected rock samples were crushed in a jaw crusher and a roll mill, and sieved. Whole rock splits were taken after crushing the kg-sized samples. White mica concentrates were obtained by using a vibrating table, repeated grinding in ethanol and sieving, magnetic purification on a Frantz isodynamic magnetic separator and washing in acetone. Subsequently impurities were removed by handpicking under an optical microscope, increasing the optical purity to > 99%. Different well-defined primary grain size- and magnetic-fractions were produced (see Table 3).

**Table 3:** ID-TIMS Rb/Sr analytical results for the Permian metapegmatites and the well-defined grain size- and magnetic-fractions of muscovite Wm I. FeO-concentrations are the mean of up to 50 EPMA-analyses from the embedded aliquots. A Frantz isodynamic magnet separator with chute inclinations relative to horizontal of 10–13° was used for magnetic purification (n.m. = not measured; nm = non-magnetic at □Volts; m = magnetic at □Volts; Rb–Sr muscovite–whole rock apparent ages have been calculated with a decay constant of 1.42 ∗ 10^− 11^ a^− 1^). From top to bottom, samples are ordered by deformation intensity.

Sample no.	Material	FeO [wt.%] (n = 50)	Rb [ppm]	Sr [ppm]	^87^Rb/^86^Sr	^87^Sr/^86^Sr	2σ error on ^87^Sr/^86^Sr	Wr–Ms age in Ma		2σ
*Meta-pegmatite*										
M1210										
TE-F0	Whole-rock		184	18.30	29.7	0.938380	0.000007			
TE-F1	Ms 250–450 μm; nm 28 V	1.39	737	2.03	1525	5.343540	0.000034	207.2	±	2.1
TE-F2	Ms 250–450 μm; m 28 V	n.m.	747	2.55	1230	5.283590	0.000060	254.6	±	2.6
TE-F3	Ms > 450 μm; m 32 V	1.42	739	1.68	1925	5.917890	0.000073	184.8	±	1.8
TE-F4	Ms > 450 μm; nm 42 V	1.39	740	1.91	1652	5.560900	0.000089	200.3	±	2.0
TE-F5	Ms > 450 μm; m 42 V	1.41	723	1.61	1954	5.807510	0.000040	177.9	±	1.8

*Meta-pegmatite*										
M1217										
TE-B0	Whole-rock		143	32.67	12.8	0.775608	0.000004			
TE-B1	Ms 250–450 μm; nm 28 V	1.75	683	4.56	504	2.360024	0.000021	226.8	±	2.3
TE-B2	Ms 250–450 μm; m 28 V	n.m.	659	5.45	398	2.098390	0.000012	241.6	±	2.4
TE-B3	Ms > 450 μm; m 32 V	2.13	688	4.51	514	2.399020	0.000033	227.6	±	2.3
TE-B4	Ms > 450 μm; nm 42 V	1.69	699	4.24	562	2.524294	0.000033	223.7	±	2.2
TE-B5	Ms > 450 μm; m 42 V	1.95	691	4.98	470	2.468850	0.000061	260.2	±	2.6

*Meta-pegmatite*										
HM00305										
TE-A0	Whole-rock		115	8.10	41.5	0.850000	0.000024			
TE-A1	Ms > 250 μm (125–250); m 40 V	n.m.	504	1.61	1316	5.358590	0.000040	248.8	±	2.5
TE-A2	Ms > 250 μm (100–125); nm 40 V	n.m.	521	1.47	1528	5.720270	0.000066	230.3	±	2.3
TE-A3	Ms > 250 μm (125–250); nm 40 V	n.m.	514	1.22	1913	6.484900	0.000132	211.7	±	2.1

*Meta-pegmatite*										
M1203										
TE-C0	Whole-rock		104	50.88	5.95	0.750029	0.000003			
TE-C1	Ms 250–450 μm; nm 28 V	1.74	468	3.18	476	1.909100	0.000079	173.5	±	1.7
TE-C2	Ms 250–450 μm; m 28 V	2.25	463	4.21	352	1.787880	0.000014	210.8	±	2.1
TE-C3	Ms > 450 μm; m 32 V	2.25	470	4.77	317	1.843320	0.000025	246.9	±	2.5
TE-C4	Ms > 450 μm; nm 42 V	1.83	481	3.50	449	2.014660	0.000024	200.9	±	2.0
TE-C5	Ms > 450 μm; m 42 V	1.93	473	3.58	428	1.910080	0.000056	193.5	±	1.9

*Meta-pegmatite*										
M1201										
TE-E0	Whole-rock		91	54.11	4.86	0.747920	0.000005			
TE-E1	Ms 250–450 μm; nm 28 V	1.54	503	4.70	339	1.675110	0.000002	195.0	±	1.9
TE-E2	Ms 250–450 μm; m 28 V	n.m.	489	4.35	354	1.627210	0.000024	177.0	±	1.8
TE-E3	Ms > 450 μm; m 32 V	1.51	519	3.32	508	1.944220	0.000026	167.4	±	1.7
TE-E4	Ms > 450 μm; nm 42 V	1.45	526	3.61	474	1.975890	0.000004	184.1	±	1.8
TE-E5	Ms > 450 μm; m 42 V	1.53	495	2.98	542	1.989970	0.000020	162.7	±	1.6

*Meta-pegmatite*										
M1206										
TE-D0	Whole-rock		143	54.40	7.64	0.776630	0.000004			
TE-D1	Ms > 250 μm; nm 20 V	n.m.	927	8.88	329	1.627490	0.000015	186.0	±	1.9

For isotope dilution analysis, 100–200 mg of each muscovite concentrate was weighed into Savillex® screw top beakers, mixed with a ^87^Rb–^84^Sr spike and dissolved in a 4:1 HF/HNO_3_ mixture on a hot plate for 2 weeks at 110 °C. Rb and Sr were extracted applying standard cation exchange techniques. Sr isotope ratios were measured on a FINNIGAN® Triton multicollector thermal ionisation mass spectrometer. Within-run mass-dependent Sr isotope fraction was corrected for with ^86^Sr/^88^Sr = 0.1194. The ^87^Rb/^85^Rb ratio was measured on a FINNIGAN® MAT262 mass spectrometer. Both instruments are part of the Laboratory of Geochronology at the University of Vienna. During the 9-month measuring period, the ^87^Sr/^86^Sr value of the NBS standard SRM 987 was 0.710269 ± 4 (2σ, *n* = 17). Total procedural blanks of Rb and Sr were consistently below 0.03 ng. By default, a 1% relative error is assigned to the ^87^Rb/^86^Sr ratio. All other errors are quoted on the 2σ level (95% confidence). Age calculations are based on a ^87^Rb decay constant of 1.42 ∗ 10^− 11^ a^− 1^ (Steiger & Jäger, 1977). A second aliquot of each muscovite concentrate was used for the production of a mineral separate embedded in epoxy resin. Polished thin sections prepared from the embedded separates were used for major element compositional analysis with the electron microprobe.

### 3.3 Major and trace element analyses by ICP-OES/MS

A third aliquot of separated muscovite fractions and the whole rocks was used for major and trace element analyses by ICP-OES and ICP-MS. Selected elements (K, Na, Ca, P, Be, Li, Cs, REE) were measured on an Agilent 7700 Series inductively coupled mass spectrometer (ICP-MS) and a PerkinElmer Optima 5300 DV inductively coupled optical emission spectrometer instrument (ICP-OES) at the Department of Environmental Sciences at the University of Vienna. REE was exclusively analysed by ICP-MS. Standards were run at the end of each measuring cycle. The reproducibility of the standards is generally better than 20%.

## 4 Results

### 4.1 Sample description

**Fig. 2:**
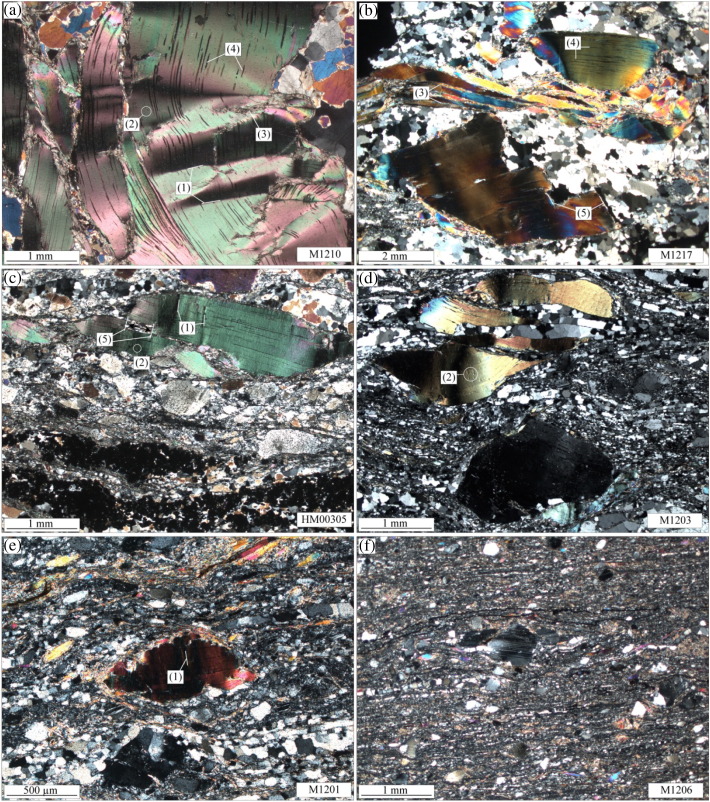
Representative microstructures of (a) M1210, (b) M1217, (c) HM00305, (d) M1203, (e) M1201 and (f) M1206. Note the intense grain-size reduction of clasts and in the matrix with increasing deformation intensity from (a) to (f). Numbers in round brackets highlight specific microstructures discussed in the text.

For the isotopic and compositional analyses, 6 samples from four different pegmatite outcrops have been selected (Table 1). Four of them stem from a single proto- to ultra-mylonitic meta-pegmatite layer (Fig. 1c) of about 1.5 m thickness and extending laterally over > 100 m. Deformation intensity increases across this meta-pegmatite layer towards the footwall (Fig. 1c). The latter also shows pronounced grain-size reduction especially of albite, K-feldspar, muscovite and – if present – garnet (Fig. 2). The remaining two were sampled from two different pegmatite bodies about 25 m (sample M1217) and 450 m (sample M1210) structurally above the former outcrop, estimated approximately normal to the mylonitic foliation. Sample M1210 stems from the centre of a massive, largely undeformed metapegmatite body and M1217 was collected near the core of a massive, weakly foliated metapegmatite. Whereas samples M1210 and M1217 stem from positions at some distance from a Cretaceous shear zone, the remaining samples HM00305, M1203, M1201 and M1206 stem from a Cretaceous high strain zone localised within meta-pegmatite at the lithological boundary between Bt–Sill gneiss and orthogneiss (Fig. 1, Fig. 2).

#### 4.1.1 Muscovite

Permian magmatic relic muscovite generally occurs as cm- to mm-sized porphyroclasts, with decreasing grain-size from the undeformed metapegmatite M1210 (> 1 cm) to the ultramylonite M1206 (< 500 μm; Fig. 2a–f). Several samples show prominent kinks with kink band widths of several hundred μm ((1) in Fig. 2a,c,e). Additionally, muscovite clasts in several samples contain numerous microkinks with lengths of < 20–30 μm and slight lattice bendings as displayed by undulose extinction in polarised light microscope images ((2) in Fig. 2a,c,d). According to Bell et al., (1986), kinks are generally associated with dislocations and cleavage cracks parallel to muscovite (001). The strong lattice deformation along the kink axial planes is associated with a fine-grained (< 50 μm) muscovite generation ((3) in Fig. 2a,b). Additionally in the deformed samples, fine-grained muscovite is also present in the quartzo-feldspathic rock matrix, defining a weak to strong mylonitic foliation by its shape preferred orientation (Fig. 2c–f). However, in none of the samples a correlation between clast grain-size and presence/absence of large kinks or microkinks was observed. Coarse-grained clasts in a few samples (M1210 and M1217) contain numerous, μm-sized, acicular inclusions of Fe-oxides and -sulphides with a clear shape preferred orientation parallel to muscovite (001) ((4) in Fig. 2a,b) and small (< 20 μm), euhedral apatite crystals. In none of the samples, a spatial correlation between presence/absence of such inclusions and kinks of any size was found. Several clasts show evidence for grain-scale cracking at high angles to muscovite (001) ((5) in Fig. 2b,c).

#### 4.1.2 Quartz

Quartz microstructures indicate recovery by subgrain rotation and grain boundary migration recrystallisation, with decreasing grain-size of the recrystallised quartz grains from the largely undeformed metapegmatite M1210 to the ultramylonite. The quartz grain-size decreases from a few hundred μm in M1210 to < 40 μm in M1206 (Fig. 2). Assuming constant temperatures during the Cretaceous tectonometamorphic event, this is explained by substantially higher strain-rates in the ultramylonitic sample M1206, compared to M1210 (cf. Stipp et al., 2002). Additionally, the mylonitic foliation, as expressed by the shape preferred orientation of the small muscovite generation, is also preserved in a weak shape preferred orientation of quartz.

#### 4.1.3 Feldspar

Albite and K-feldspar have survived the intense Cretaceous mylonitisation as clasts that range in size from > 1 cm in M1210 to < 500 μm in M1206 (Fig. 2). Furthermore, both feldspars preserve evidence for extensive dissolution–precipitation, producing grain-sizes of < 50 μm, that tend to decrease from M1210 to M1206. Together with alternating layers of quartz, muscovite and fractured garnet (< 100 μm), these delineate a mylonitic foliation in the high-strain samples (e.g., Fig. 2c–f).

### 4.2 Muscovite generations and mineral assemblages

**Fig. 3:**
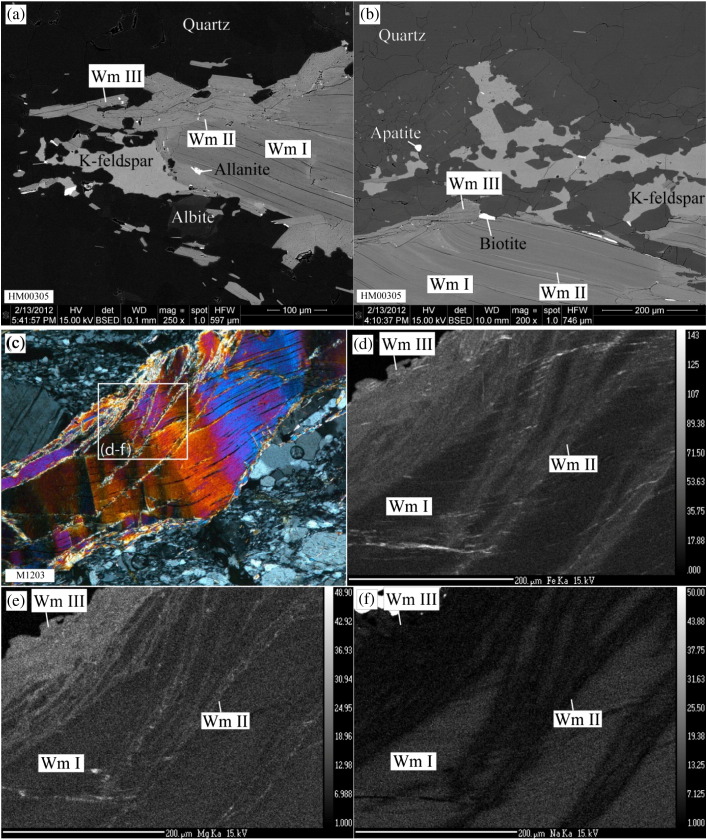

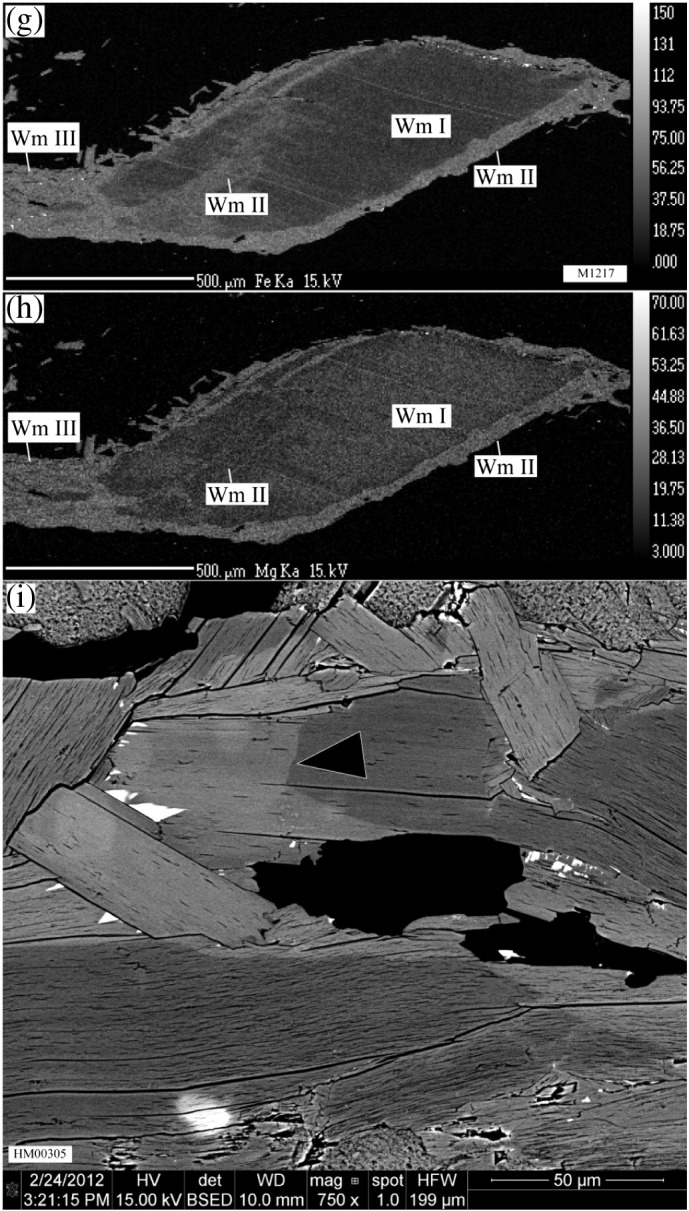
(a–b) BSE images of sample HM00305 showing the different muscovite groups (Wm I–III), their inclusions and the related syntectonic phase assemblage including WM III. (c) Photomicrograph with crossed polarised light showing a kinked and fractured Ms-clast of sample M1203. (d–f) Quantitative element maps showing (d) Fe- ,(e) Mg- and (f) Na-distribution in the area highlighted in panel (c). (g–h) Quantitative element maps of a mm-sized Permian muscovite clast from sample M1217 showing (g) Fe- and (h) Mg-distribution related to the different muscovite groups. Note, the weak compositional difference between Wm II associated with the clast-internal domain (crack) and the Wm II along the rims, possibly representing different generations of Wm II. (i) Detailed BSE-image of a sharp transition front between Wm I and Wm II (head of black arrow) in sample HM00305.

Based on backscatter electron images and element mapping 3 distinct groups of white mica have been identified in all samples (Fig. 3). Core-domains of coarse-grained Permian magmatic muscovite clasts have unaltered primary major element composition (Wm I). These have compositionally altered rims (Wm II) which are separated from Wm I by compositional fronts with sharp transitions (e.g., Fig. 3i). Wm II is microstructurally not distinguishable from Wm I, but may also form alteration zones within the interior of coarse-grained clasts. Wm II occurs immediately adjacent to (001) cleavage planes (Fig. 3b) and along fractures (Fig. 3c–h). The microstructurally characteristic fine-grained (< 50 μm) third muscovite group (Wm III) constitutes the mylonitic rock matrix, forms overgrowths on Wm I (Fig. 3a–b) and newly crystallised along kink axial planes.

Qualitative EDX analysis in SEM in combination with BSE imaging showed that μm-sized inclusions of allanite, biotite and apatite are often related with the compositionally altered rims Wm II of the Permian muscovite clasts Wm I (Fig. 3a,b). Furthermore, the syntectonic phase assemblage muscovite (Wm III), fine-grained K-feldspar, biotite, apatite, albite and quartz adjacent to the muscovite clasts has been identified in the mylonitic rock matrix and in strain shadows of Wm I clasts (Fig. 3a,b).

### 4.3 Mineral composition

#### 4.3.1 Muscovite

Representative compositions of muscovite groups Wm I, Wm II and Wm III derived from EMPA are given in Table 2.

##### 4.3.1.1 Wm I — Permian magmatic muscovite

**Fig. 4:**
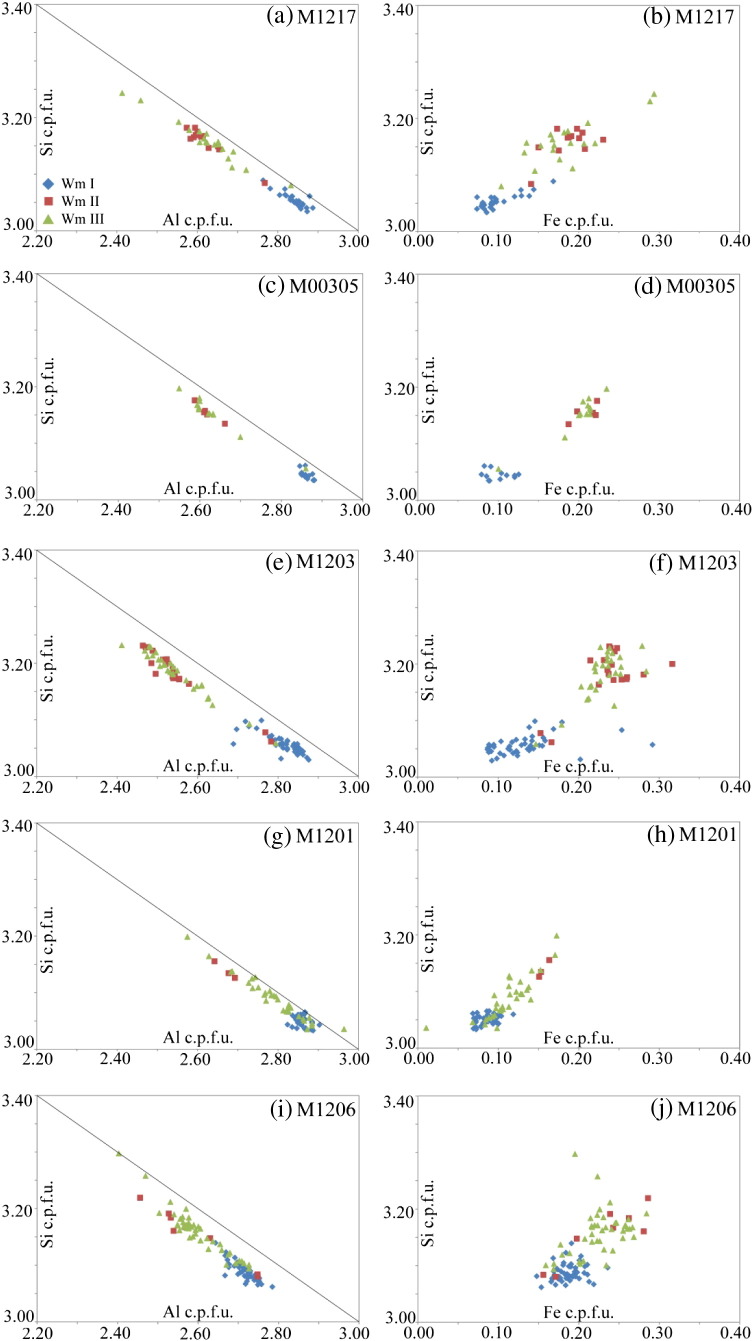
Muscovite compositional data by EMPA of Wm I (blue diamonds), Wm II (red squares), Wm III (green triangles) of (a–b) M1217, (c–d) HM00305, (e–f) M1203, (g–h) M1201 and (i–j) M1206. Note the compositional overlap of different generations in the high-strain samples M1201 and M1206. For legend, see panel a.

The cm-sized Permian magmatic muscovite grains are compositionally close to the pure muscovite endmember with < 3.1 Si c.p.f.u. (cations per formula unit), > 2.7 Al c.p.f.u., and commonly less than 0.2 Fe^2 +^ c.p.f.u. and < 0.04 Mg^2 +^ c.p.f.u. (Fig. 4, Table 2).

##### 4.3.1.2 Wm II — compositionally overprinted rims of Wm I

Wm II portions of coarse grained clasts are commonly richer in Fe with Fe^2 +^ c.p.f.u. in the range 0.15–0.3. Si typically ranges from 3.1 to 3.2 Si c.p.f.u. with some exceptions of Si c.p.f.u. > 3.2 (Fig. 4, Table 2). Wm II and Wm III are compositionally identical (Fig. 4). However, small compositional fluctuations within Wm II may also exist. An example for this is displayed by one mm-sized muscovite clast in sample M1217. Here, a grain-scale crack in that clast is associated with Wm II that is slightly poorer in Fe and Mg, compared to Wm II on the clasts rim (Fig. 3g–h). Wm II forms 10–40 micrometre thick rims of Wm I and is separated from the latter by sharp compositional fronts (e.g., Fig. 3i).

##### 4.3.1.3 Wm III — matrix muscovite

Mylonitic matrix muscovite is often compositionally indistinguishable from Wm II (Fig. 3, Fig. 4, Table 2). In all medium- and low-strained samples, Wm II and Wm III have significantly higher Al-celadonite contents (elevated Fe and Si contents) than Wm I allowing for a clear distinction between primary magmatic muscovite (Wm I) and muscovite-fractions formed during the metamorphic overprint (Wm II, Wm III). Contrastingly, the highly strained samples M1201 and M1206 display a wider scatter of the Wm II and Wm III compositions, causing an overlapping compositional range of the microstructurally different muscovite generations (Fig. 4).

#### 4.3.2 Embedded muscovite separates

**Fig. 5:**
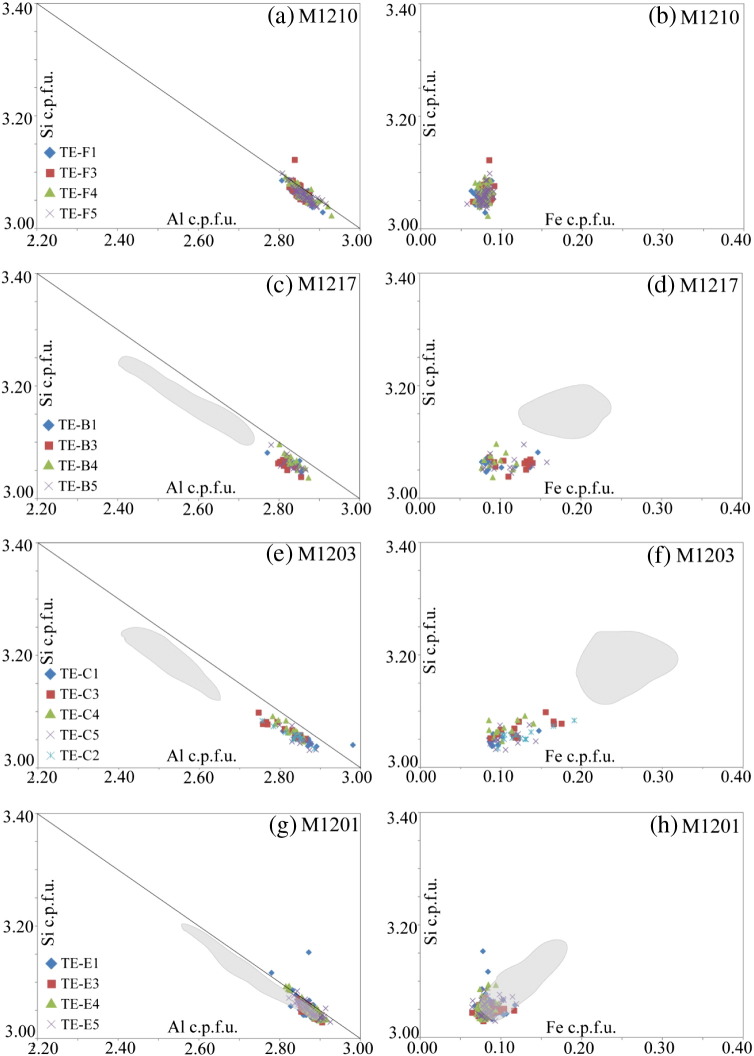
EMPA compositional data of embedded muscovite separates of Wm I used for Rb/Sr ID-TIMS analysis of (a–b) M1210, (c–d) M1217, (e–f) M1203 and (g–h) M1201. Grey areas limit the compositional range of Wm II and Wm III determined by EMPA from thin sections of rock chips. Note the overlap of this field with data from Wm I separates in M1201 (g–h) (cf. Fig. 4). Sample HM00305 was not analysed as the separated Wm I did not provide enough material for an additional aliquot. See text for discussion.

Muscovite separates from samples M1210, M1217, M1203 and M1201 were embedded in epoxy resin and analysed by EMPA in order to detect a potential contribution of the metamorphic muscovite fractions (Fig. 5). The EMPA data from embedded separates showed that all different muscovite grain size and magnetic fractions consist exclusively of Wm I (Fig. 5). The embedded material is characterised by relatively low Si (< 3.1 Si c.p.f.u.) and low Fe (< 0.15 Fe^2 +^ c.p.f.u.). Only in the high-strain sample M1201, there is a compositional overlap with Wm II and Wm III as indicated by the grey areas that are shown to highlight the compositional range of these 2 groups which have been derived from grains during thin section analyses (Fig. 5). Contributions from other phases and sub-microscopic mineral inclusions were not observed in the embedded muscovite separates, although a potential contribution of sub-μm sized feldspar, apatite or zoisite/allanite inclusions can never be explicitly excluded. The mean FeO concentrations of up to 50 single spot analyses of individual grains for each fraction are given in Table 2. Some data show a weak correlation of FeO concentration with magnetic susceptibility, thus pointing to the role of Fe in controlling the magnetic properties of the analysed material. For sample HM00305, there was only sufficient muscovite material for isotopes, leaving nothing for further EPMA analysis.

#### 4.3.3 Apatite and feldspar

Sample HM00305 contains fine-grained (< 20 μm) euhedral apatite crystals, spatially related to the syntectonic phase assemblage (Fig. 3a–b). Some of these supposedly Cretaceous apatite crystals are rich in fluorine (1.1–3.3 wt.% F), poorer in chlorine (< 0.4 wt.% Cl) and contain small concentrations of Sr (~ 0.01 wt.% SrO) as determined by electron microprobe analysis. Albite grains from a different microdomain within the same thin section formed by dissolution–precipitation and also contain some Sr (~ 0.02–0.05 wt.% SrO).

### 4.4 Isotope, major and trace element data (ICP-MS and ID-TIMS)

#### 4.4.1 Permian metapegmatite bulk compositions

**Fig. 6:**
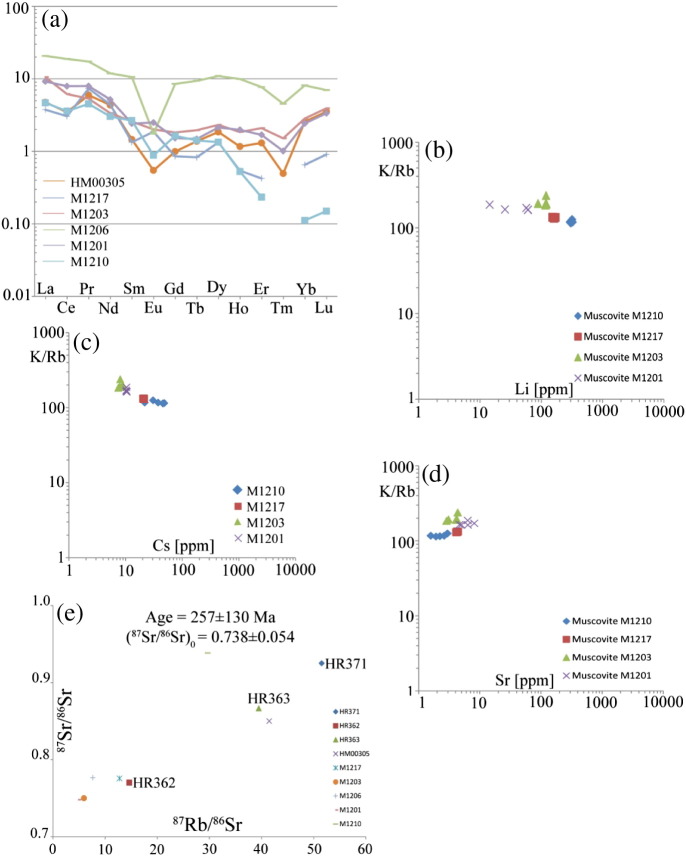
Compositional characteristics of Permian pegmatites and magmatic muscovites. (a) Chondrite-normalised REE pattern of the pegmatites (CI-values from Sun & McDonough, 1989), (b) K/Rb vs Li, (c) K/Rb vs Cs and (d) K/Rb vs Sr of the muscovites and (e) Rb–Sr whole rock data of the pegmatites. Data labelled HR371, HR362 and HR363 are from Haas (1985).

Permian metapegmatites are Si-rich rocks (> 73 wt.% SiO_2_) with moderate Al-concentrations (12.5–15 wt.% Al_2_O_3_). Their K/Rb ratios are 130–250, while Rb/Cs ratios range at 15–80. At Li-concentrations of 16–30 ppm their REE-contents are generally below 1–2 ppm. In M1203, M1201 and M1206, the concentrations of La, Ce and Nd are slightly elevated (up to 11.5 ppm, Table 4). Their chondrite-normalised REE-pattern shows no significant fractionation (Ce_n_/Yb_n_ < 5) except the weakest deformed sample M1210 (TE-F0) in which Ce_n_/Yb_n_ = 32 (Fig. 6a). The ultramylonite sample M1206 has REE-concentrations about one order of magnitude larger than the other samples. Apart from that, no relation between intensity of deformation and REE-contents in the bulk samples was observed. Some samples exhibit a weak negative Eu-anomaly (HM00305, M1210, M1206), while the other samples do not show significant anomalies (Fig. 6a).

**Table 4:** Major and trace element analyses of muscovite fractions and whole rocks as determined by ICP-OES and ICP-MS. Major elements are given in wt.% and trace elements are ppm.

Sample	HM00305	M1217				M1203					M120	M1201					M1210					HM00305	M1217	M1203	M1206	M1201	M1210
	TE-A3	TE-B1	TE-B3	TE-B4	TE-B5	TE-C1	TE-C2	TE-C3	TE-C4	TE-C5	TE-D1	TE-E1	TE-E2	TE-E3	TE-E4	TE-E5	TE-F1	TE-F2	TE-F3	TE-F4	TE-F5	TE-A0	TE-B0	TE-C0	TE-D0	TE-E0	TE-F0
Type	Muscovite																					Whole rocks					
CaO	0.03	0.03	0.03	0.03	0.03	0.03	0.04	0.04	0.03	0.03	0.08	0.14	0.06	0.04	0.07	0.05	0.03	0.04	0.07	0.04	0.04	0.51	0.38	0.48	0.65	0.54	0.18
Na_2_O	0.60	0.47	0.48	0.48	0.51	0.49	0.45	0.52	0.56	0.51	0.53	0.58	0.55	0.61	0.60	0.64	0.55	0.55	0.61	0.56	0.59	5.50	4.13	4.85	3.89	4.28	2.87
K_2_O	10.37	10.67	10.76	10.89	10.80	10.82	10.90	10.80	11.04	14.04	11.17	10.82	10.86	10.72	10.69	10.70	10.96	10.56	11.21	10.82	11.08	2.25	2.96	3.32	2.04	2.56	6.87
P_2_O_5_	0.02	0.02	0.02	0.02	0.02	0.02	0.02	0.02	0.03	0.02	0.03	0.02	0.03	0.02	0.03	0.02	0.02	0.03	0.02	0.03	0.02	0.15	0.12	0.11	0.22	0.10	0.18
Be	9.56	76.70	75.45	75.32	76.96	28.09	24.20	26.44	27.35	25.87	55.02	55.07	57.00	44.21	40.14	45.82	56.51	57.50	55.70	59.52	56.42	5.55	65.22	31.31	69.83	53.03	114.51
Li	67.17	152.60	163.80	169.99	161.38	117.42	88.27	121.34	117.15	119.85	121.47	57.05	14.37	61.03	25.64	60.47	305.11	292.63	317.33	302.88	324.21	16.65	30.05	24.72	24.04	15.88	27.74
Cs	6.33	20.99	21.23	20.39	20.79	8.51	9.08	7.90	7.46	8.07	17.94	10.32	10.41	10.62	10.26	10.49	48.28	21.82	30.64	45.38	37.50	3.28	6.80	2.28	1.73	1.72	26.79
Rb	491.53	667.95	684.98	683.12	693.83	473.92	471.47	472.73	498.89	489.33	913.80	527.29	483.80	546.07	540.62	547.79	793.13	752.92	743.47	790.78	787.05	89.79	136.68	100.97	133.18	85.91	383.13
La	0.09	0.10	0.07	0.10	0.06	0.25	0.29	0.21	0.20	0.26	0.97	0.24	0.24	0.12	0.15	0.16	0.05	0.16	0.08	0.05	0.05	1.14	0.90	2.53	4.97	2.21	1.12
Ce	0.03	0.06	0.05	0.06	0.08	0.17	0.40	0.09	0.17	0.26	1.64	0.42	0.48	0.24	0.77	0.44	0.26	0.92	0.17	0.12	0.11	2.11	1.86	3.76	11.48	4.83	2.18
Pr	bdl	bdl	bdl	bdl	0.05	0.02	0.42	bdl	0.12	0.14	0.41	0.10	0.34	0.16	0.61	0.24	0.10	0.80	0.07	0.13	0.08	0.54	0.66	0.48	1.56	0.71	0.41
Nd	0.17	0.29	bdl	0.02	0.38	0.15	1.03	0.22	0.45	0.48	1.16	0.44	1.31	0.67	2.23	0.68	0.60	3.22	0.30	0.43	0.38	2.00	2.03	1.54	5.46	2.38	1.39
Sm	0.02	0.02	0.04	bdl	0.01	0.04	0.07	0.05	0.04	0.05	0.20	0.01	0.06	0.02	0.02	0.02	0.03	bdl	0.02	bdl	bdl	0.22	0.20	0.39	1.59	0.36	0.40
Eu	bdl	0.01	0.01	0.01	0.01	0.01	0.03	0.01	0.01	0.02	0.02	0.02	0.03	0.01	bdl	0.02	0.01	bdl	0.01	bdl	0.01	0.03	0.11	0.12	0.11	0.15	0.05
Gd	0.02	bdl	bdl	bdl	0.01	0.03	0.03	0.02	0.02	0.01	0.25	0.01	0.03	0.02	0.02	0.01	bdl	0.08	0.02	bdl	0.02	0.20	0.17	0.36	1.70	0.30	0.33
Tb	bdl	bdl	bdl	bdl	0.01	bdl	bdl	bdl	bdl	bdl	0.04	bdl	bdl	bdl	bdl	bdl	bdl	bdl	bdl	bdl	bdl	0.06	0.03	0.08	0.38	0.06	0.06
Dy	0.06	0.05	0.06	0.07	0.13	0.09	0.20	0.09	0.11	0.10	0.34	0.09	0.16	0.07	0.27	0.10	0.07	0.15	0.08	0.05	0.05	0.46	0.33	0.58	2.73	0.53	0.33
Ho	bdl	bdl	bdl	0.01	0.01	bdl	bdl	bdl	bdl	bdl	0.04	bdl	bdl	bdl	bdl	0.01	bdl	bdl	bdl	bdl	bdl	0.06	0.03	0.09	0.50	0.10	0.03
Er	bdl	bdl	bdl	0.01	0.02	0.01	0.03	0.02	0.01	0.01	0.14	0.01	0.03	0.01	0.02	0.04	bdl	bdl	bdl	0.01	bdl	0.21	0.07	0.33	1.23	0.27	0.04
Tm	bdl	bdl	bdl	bdl	bdl	bdl	bdl	bdl	bdl	bdl	bdl	bdl	bdl	bdl	bdl	bdl	bdl	bdl	bdl	bdl	bdl	0.01	bdl	0.03	0.09	0.02	bdl
Yb	bdl	bdl	bdl	0.02	0.01	0.03	bdl	0.01	0.02	0.01	0.08	0.02	0.03	0.02	bdl	0.03	bdl	bdl	bdl	bdl	bdl	0.41	0.10	0.45	1.30	0.39	0.02
Lu	bdl	bdl	bdl	bdl	bdl	0.01	bdl	bdl	bdl	bdl	0.02	bdl	bdl	bdl	bdl	bdl	bdl	bdl	bdl	bdl	bdl	0.07	0.02	0.08	0.14	0.07	bdl

Concerning the composition of different muscovite fractions, their Li- and Cs-contents are increasing with decreasing K/Rb ratios (Fig. 6b–c), while with increasing Sr-concentrations the K/Rb also increases (Fig. 6d). The Sr-concentration of muscovite from sample M1210 are slightly lower than in the remainder of the investigated samples, which is consistent with the significantly lower Sr-concentration of the corresponding whole rock (18.3 ppm in M1210, Table 2) and resulting in a higher ^87^Rb/^86^Sr ratio of 29.7. Sample HM00305 shares similar compositional and isotopic features with M1210 (Table 4) having lower Sr-concentrations and consequently higher ^87^Rb/^86^Sr and ^87^Sr/^86^Sr ratios compared to the other samples. The ^87^Sr/^86^Sr ratios of 0.93838 (M1210) and 0.85000 (HM00305) are significantly larger compared to the other samples, which range from 0.74792 to 0.77663 (Table 4, Fig. 6e). Calculating a Rb–Sr whole rock regression using all samples from the current study and the data of the samples HR371, HR362 and HR363 from Haas (1985), results in a poorly defined apparent age of 257 ± 130 Ma (Fig. 6e). Despite the common caveats, like isotopic inhomogeneity (e.g., Brooks et al., 1968) and the huge error, the result is still consistent with a pegmatite forming event in the Permo-Triassic (cf. Haas, 1985).

#### 4.4.2 Muscovite

**Fig. 7:**
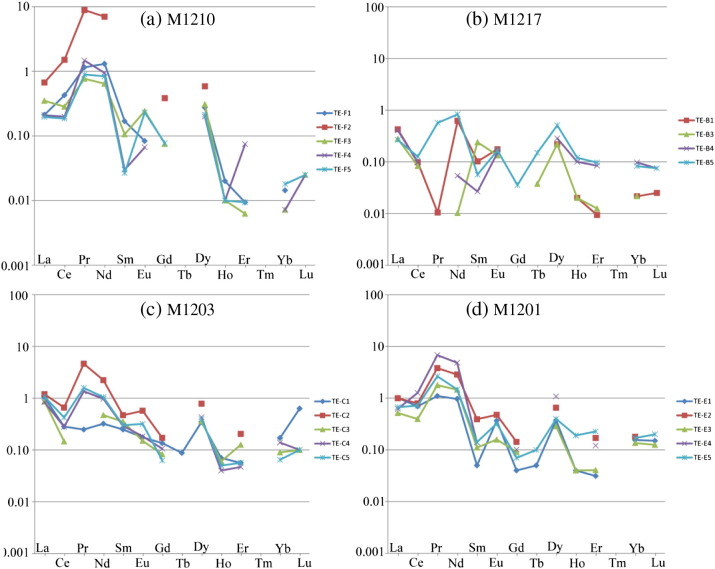
Chondrite-normalised REE-pattern of the different muscovite fractions.

With the exception of Nd, muscovite has REE contents of < 1 ppm (Table 4). Despite these low concentrations, the chondrite-normalised REE-pattern of the different muscovite fractions shares the following similarities (Fig. 7): (i) there is almost no REE-fractionation, (ii) positive Pr–Nd–(Eu–)Dy–anomalies are recognised in all samples and (iii) no correlation between deformation intensity and REE-pattern or -concentration exists (Fig. 7). Additionally, no correlation between REE-pattern and Rb–Sr muscovite–whole rock ages can be identified (cf. Fig. 7 and Table 2, Table 4).

**Fig. 8:**
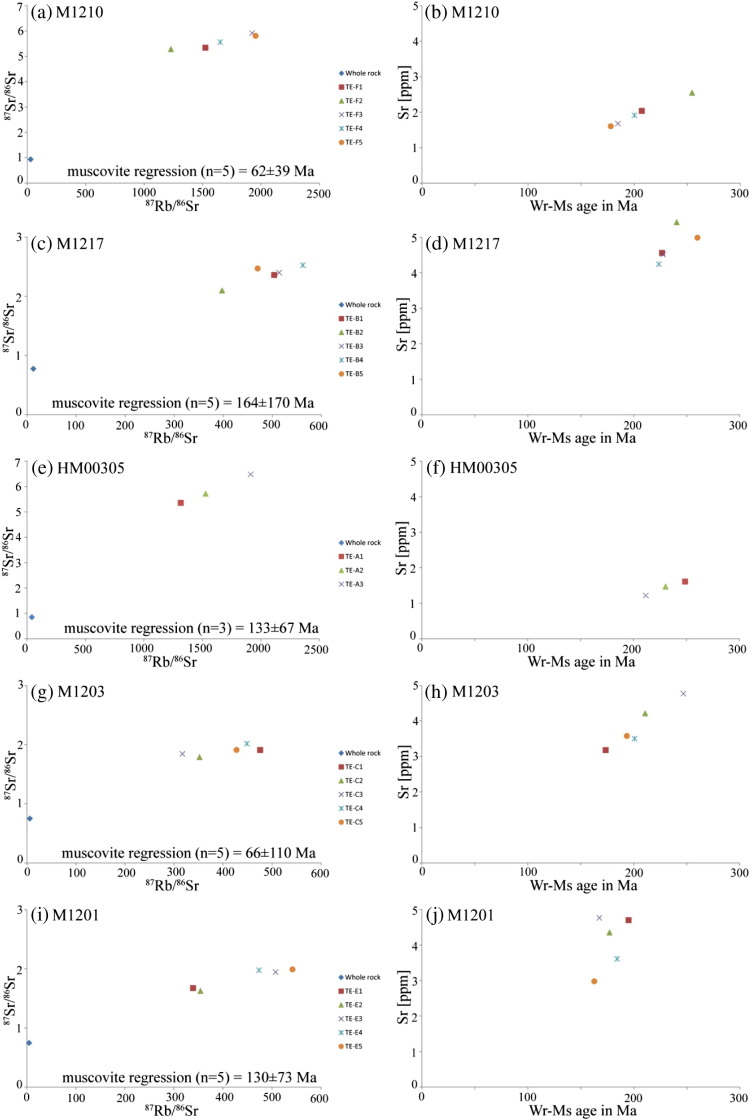
ID-TIMS Rb/Sr data plotted in ^87^Rb/^86^Sr vs ^87^Sr/^86^Sr space (left column) and Sr-concentrations vs Rb–Sr muscovite–whole rock apparent age (right column) for (a–b) M1210, (c–d) M1217, (e–f) HM00305, (g–h) M1203 and (i–j) M1201. Age values in the left column refer to muscovite-internal regressions. Note the scatter of these around the time of the Cretaceous event and the positive correlation trend of Sr in muscovite with the in muscovite–whole rock apparent age.

**Fig. 9:**
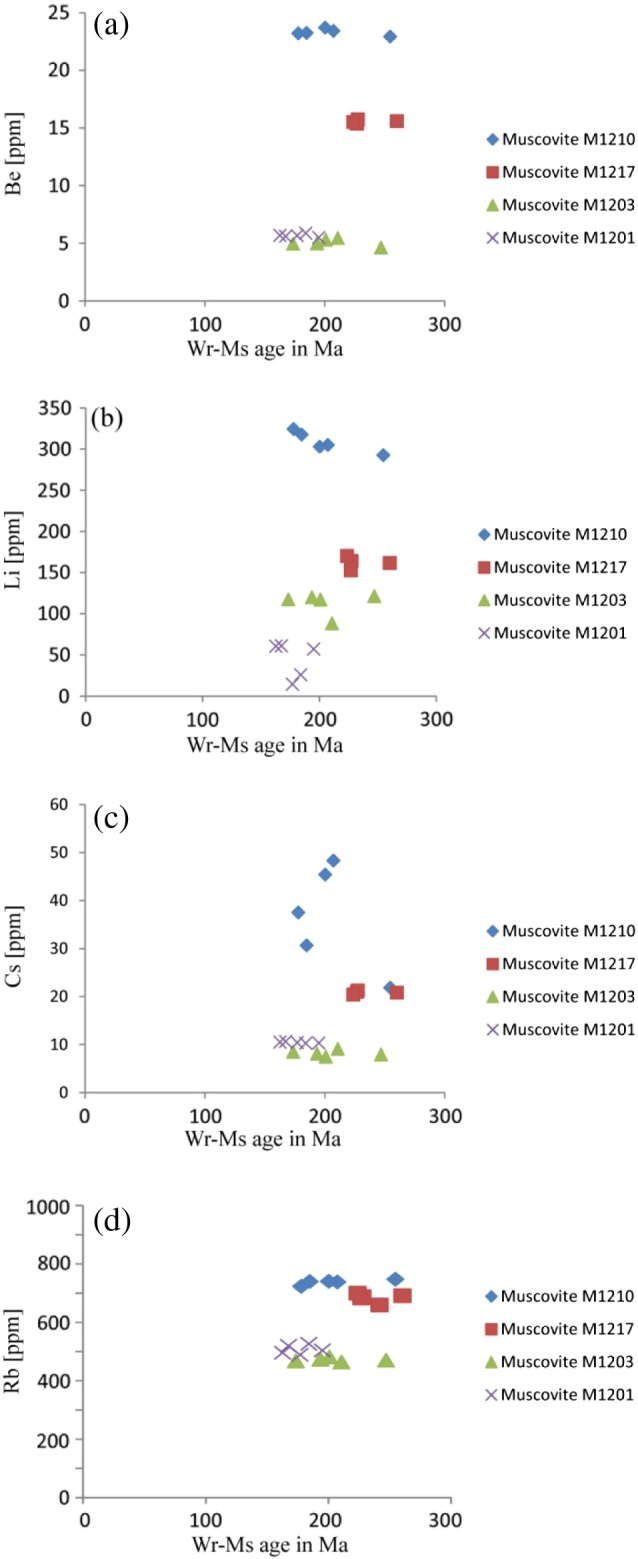
Plots of (a) Be-, (b) Li-, (c) Cs- and (d) Rb-concentrations vs Rb–Sr muscovite–whole rock apparent age. Note, that none of these elements show a positive correlation with the mineral–whole rock age.

Rb-concentrations among the different muscovite fractions of single samples show only small fluctuations (Fig. 9d, Table 3), like in M1210, in which the difference in Rb-concentration between the fractions with the highest and lowest Rb-concentrations is ~ 24 ppm, at total concentrations of > 700 ppm. In comparison, Sr concentrations often vary by more than 20% relative. For example, in M1210, the difference between the grain-size and magnetic fractions TE-F2 and TE-F5 is 0.94 ppm, which is, at these concentration levels, a deviation of 36% relative (Table 3). Consistent with the whole rock data (see Section 4.4.1), muscovite from samples M1210 and HM00305 is slightly different than those from all other samples. Firstly, they have significantly lower Sr-concentrations of < 2.5 ppm, compared to the other samples with Sr-concentrations of 3–8 ppm. Secondly, their ^87^Rb/^86^Sr ratios at 1200–2000 and ^87^Sr/^86^Sr ratios at 5.2–6.5 are correspondingly larger than those from muscovite fractions of the other samples (Table 3). ICP data of additional aliquots of Wm I were used to carefully check potential contributions of apatite and/or feldspar by monitoring P (for apatite) and Ca and Na (for feldspar). In no case, we found any correlation pointing to significant contributions of such phases (see Table 4). Despite this fact, the distinct muscovite fractions of 5 samples with several muscovite grain size- and magnetic-fractions share the following common features (Fig. 8; Table 3): (i) different muscovite fractions within single samples show a positive correlation of ^87^Rb/^86^Sr and ^87^Sr/^86^Sr, with (ii) poorly defined muscovite-internal regression data ranging from 62 ± 39 to 164 ± 170 Ma. (iii) All samples, except M1201 and M1206, have at least one muscovite fraction with a Permo-Triassic Rb–Sr mineral–whole rock apparent age. (iv) The remaining muscovite fractions range at lower Sr-concentrations systematically related with higher ^87^Rb/^86^Sr ratios and therefore yield systematically decreasing calculated Rb–Sr mineral–whole rock apparent ages within single samples, spanning an “age” difference of 32 Ma in sample M1201 and 76 Ma in sample M1210 at slightly increasing ^87^Sr/^86^Sr (Fig. 8, Table 3). In general, the mean Rb–Sr mineral–whole rock apparent ages of up to 5 muscovite grain-size and magnetic fractions show a weak trend to decrease from the weakly deformed sample M1217 (236 Ma) to the mylonitic sample M1201 (177 Ma).

A comparison of the FeO- and Sr-concentrations shows that the Sr concentration variations do not correlate with the FeO-content and thus with the magnetic susceptibility of the respective fraction (Table 3). No other element is recognised to steadily decrease or increase with the calculated Rb–Sr muscovite–whole rock apparent age or deformation intensity in all samples (Fig. 7, Fig. 8, Fig. 9), suggesting that the variations in Sr are decoupled from the other elements as well. Be, Li, Cs and Rb are present in appreciable concentrations (Fig. 9). Differences in the concentrations of these elements in the different muscovite fractions of single samples are small and most likely related to primary variations between different pre-Cretaceous pegmatites and different pegmatite domains. However, it is interesting to note, that, for example, M1210 with the weakest deformational imprint shows the highest Li-, Be-, Cs- and Rb-contents, while they apparently decrease with sample deformation intensity from M1217 to M1201 (Fig. 9).

## 5 Discussion

### 5.1 Permian magmatic stage

The formation of wide-spread pegmatites in the Austroalpine Unit of the European Eastern Alps has been attributed to a long-lasting HT/LP tectonometamorphic event during the Permian–Triassic (Schuster et al., 2001, Thöni et al., 2008). Sm–Nd garnet–whole rock data from Permian metapegmatites in the Upper-Austroalpine Matsch Unit point to pegmatite formation at 263–280 Ma (Habler et al., 2009). Despite potential isotopic inhomogeneity in granitic melts (e.g., Brooks et al., 1968) and the large associated error, the regression calculation using different metapegmatite Rb–Sr whole rocks yielding 257 ± 130 Ma (Fig. 6e) is in agreement with pegmatite formation in the Permo-Triassic. The major and trace element data presented in this study indicate a rather unfractionated nature of the pegmatites. Only samples M1210 and HM00305 show some minor fractionation, as indicated by weak negative Eu-anomalies and low Sr-concentrations of the whole rocks. M1210 is additionally characterised by some REE-fractionation and elevated Li-contents and high Cs-concentrations of the whole rock and of muscovite (Fig. 6, Table 4). The low Sr-concentrations and the Eu-anomalies are possibly caused by fractionation of primary apatite, zoisite and/or plagioclase in the pegmatitic melt. The low Sr-concentrations of these samples and corresponding muscovites lead to relatively high ^87^Rb/^86^Sr whole rock ratios of > 30 (compared to < 10 in the other meta-pegmatite whole rock samples) and > 1200 in muscovite, respectively. In turn, also the ^87^Sr/^86^Sr ratios are significantly higher (Figs. 6e and 8, Table 3).

### 5.2 The effect of deformation on the major and trace element compositions of muscovite

Muscovite porphyroclasts from the studied Permian metapegmatites preserve evidence for grain-internal brittle and crystal-plastic deformation as indicated by the presence of cracks, undulose extinction, (micro-)kinks and dynamic recrystallisation, especially along kink planes (Fig. 2). Despite the partly intense mylonitisation of the Permian pegmatites during the Cretaceous tectonometamorphism (Fig. 1c), primary magmatic muscovite persisted as cm- to sub-mm-sized clasts (Fig. 2). They largely retained their magmatic major element composition of nearly pure muscovite endmember, as reflected by their relatively homogeneous major element composition (Fig. 3). According to combined microstructural and mineral compositional characteristics, fine-grained matrix muscovite (Wm III) formed by new muscovite crystallisation, recrystallisation by dissolution–precipitation creep, and dynamic recrystallisation. Their formation is spatially clearly related with deformation microstructures: they represent the mylonitic foliation of the quartzo-feldspathic rock matrix; they crystallised in strain shadows of coarse-grained Ms-clasts and along cracks within Ms-clasts; and crystallisation occurred at sites of high dislocation densities like kink planes of primary clasts (Fig. 2). Significant compositional changes of muscovite occurred in relation with dynamic recrystallisation producing Wm III, especially in the mylonitic matrix and along cracks and kink planes and chemical alteration producing Wm II on rims, along cracks and muscovite (001) cleavage planes (Fig. 3c–i). Similar altered muscovite rims with sharp compositional fronts but with different compositional characteristics as our Wm II have been observed elsewhere in hydrothermally altered granites (e.g., Dempster et al., 1994, Gomes and Neiva, 2000). Dempster et al. (1994) found such secondary rims on primary magmatic muscovites from the Oughterard granite in western Ireland. They correlated increasing Si-contents (up to 3.4 c.p.f.u.) of these secondary muscovites with increasing degree of hydrothermal alteration. Gomes and Neiva (2000) presented a detailed compositional analysis of such rims from zoned muscovite in the Ervedosa granite in northern Portugal. According to their data, the overgrowth is richer in Fe + Mg and Rb but has less Al and Na, than the relic magmatic muscovite.

The normalised REE-concentrations of the muscovite fractions indicate that the REE characteristics of the differently strained samples are similar (Fig. 7). Thus, it is concluded, that the REE characteristics of muscovite largely remain unaffected by deformation. The major element characteristics of the almost pure muscovite endmember of Permian relic muscovite (Wm I) obviously remained unaffected by Cretaceous deformation in undeformed core domains (Fig. 3) and thus are interpreted to reflect the primary composition of the muscovite grains. However, although there might be a weak correlation between the concentrations of the trace elements Li, Be, Cs and Rb and total finite strain, variations in these elements are most likely caused by bulk rock compositional variations among the different pegmatite bodies and different pegmatite domains (Fig. 9). The only element that shows a trend to decrease with decreasing Rb/Sr muscovite–whole rock apparent age is Sr. However, namely in the samples M1217 and M1201, this trend is not obvious (cf. Fig. 8).

### 5.3 The effect of deformation on the Rb–Sr isotopic system of muscovite

Microstructurally relic muscovite clasts have previously been shown to reflect incomplete isotopic resetting (e.g., Glodny et al., 2008, Villa, 1998), whereas dynamic recrystallisation and (neo-)crystallisation were supposed to potentially lead to complete isotopic resetting (Freeman et al., 1997, Glodny et al., 2002, Glodny et al., 2008, Villa, 1998, Yund and Tullis, 1991). Permo-Triassic Rb–Sr muscovite–whole rock apparent rock ages of the investigated samples confirm their relict character with respect to the Rb–Sr isotopic system.

Based on theoretical considerations, deformation is expected to have an effect on the Rb–Sr geochronometer, comparable with the effect on the Ar-retentivity of muscovite (e.g., Cosca et al., 2011, Kramar et al., 2003, Mulch et al., 2002). Deformation may introduce dislocations, point defects, new grain boundaries and cracks, all of which reduce the effective diffusion domain size, which is then smaller as the initial primary grain size of the undeformed muscovite. Therefore, deformation at upper-greenschist facies P–T conditions (T ≤ 500 °C) is supposed to be the predominant factor in affecting the Rb–Sr isotopic system of microstructurally relic muscovite (cf. Freeman et al., 1997, Glodny et al., 1998, Müller et al., 1999, Villa, 1998).

Even for single hand specimen, analyses of multiple grain size- and magnetic-fractions of Wm I yielded a range of different Rb–Sr muscovite–whole rock apparent ages positively correlating with their Sr-concentration (Fig. 8, Table 3). Deformation-induced defects, such as dislocations, point defects, new grain boundaries and cracks have acted as high-diffusivity pathways, allowing the incompatible Sr to leave the crystal lattice more easily through multipath diffusion (Lee, 1995). Wm I grain size- and magnetic-fractions with the lowest Sr-concentrations and the lowest Rb–Sr muscovite–whole rock apparent ages most likely represent more strongly deformed parts of Wm I clasts. Furthermore, the Dodson theory implies that the loss of ^87^Sr controls the Rb–Sr geochronometer (Dodson, 1973). More precisely it is a net loss of Sr, potentially coupled with very restricted uptake of Sr in muscovite from the matrix reservoir with unknown isotopic composition (Fig. 8, Table 3).

**Fig. 10:**
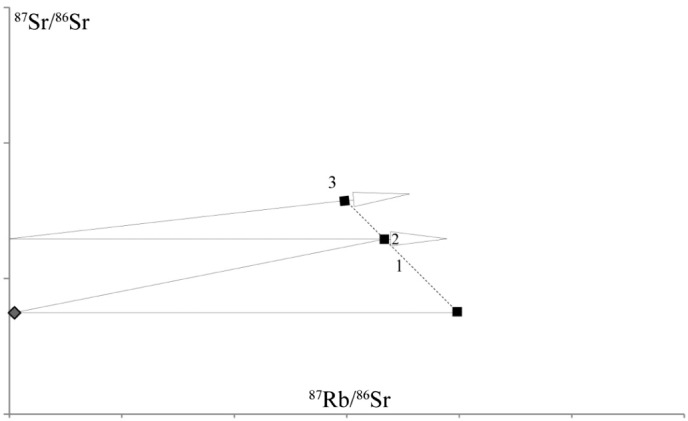
Simplified sketch representing the proposed model that includes a primary magmatic muscovite population with a homogeneous ^87^Rb/^86^Sr ratio, evolving isotope ratios (1) and a single stage net loss of Sr during the Cretaceous (2) and its predicted effect on the present-day data distribution (3).

The proposed model to explain our data assumes a pegmatitic muscovite population with an initially homogeneous ^87^Rb/^86^Sr ratio that is in isotopic equilibrium with the whole rock reservoir (Fig. 10). After the pegmatites cooled, the isotope ratios evolved along the growth line (1) (Fig. 10). The effect of the inferred net loss of Sr, potentially coupled with very restricted uptake of Sr with unknown isotopic composition from the matrix reservoir during the meta-pegmatite evolution in the Cretaceous is a spread in the ^87^Rb/^86^Sr ratios between different muscovite populations ((2) in Fig. 10). The effect on the present-day pattern is shown by the different muscovite fractions scattering around a hypothetic line ((3) in Fig. 10) with a lower slope than an undisturbed Permian muscovite–whole rock would show. The preferential loss of ^87^Sr is likely to have only minor effects as the resulting muscovite regression would yield lower apparent ages than in the proposed model (Fig. 10).

Different parts of the crystal are supposed to have lost variable amounts of Sr due to different deformation intensities, successively increasing the ^87^Rb/^86^Sr ratio of the respective muscovite population. However, as mentioned above, it is also possible, that very restricted amounts of Sr with unknown isotopic composition from the matrix reservoir may have entered the muscovite crystal lattice and thus have potentially caused also variations in the ^87^Sr/^86^Sr ratios (Fig. 10). In cases of variations in ^87^Sr loss, the influence of deformation was supposedly related with alteration by a fluid phase. The predicted present-day scattered distribution of the various muscovite populations almost matches the measured distribution of the different muscovite populations in the studied samples (cf. Fig. 8, Fig. 10). Calculating muscovite-internal regressions yield poorly defined apparent ages in our samples in the range from 62 ± 39 to 164 ± 170 Ma (cf. Fig. 8). Additionally, the presented model also allows for at least one muscovite population that neither loses nor gains Sr and thus retains its isotopic composition during the Cretaceous event (black square in Fig. 10). Consequently, a Rb/Sr muscovite–whole rock regression using this muscovite population is expected to yield the timing of Permo-Triassic formation or cooling. Indeed, except for M1201 and M1206, every sample contains at least one muscovite population with a Permo-Triassic Rb/Sr muscovite–whole rock apparent age. Furthermore, these muscovites are also those with the lowest ^87^Rb/^86^Sr ratios (Fig. 8, Table 3). Therefore, we interpret these as to reflect Permo-Triassic formation/cooling. At least, these are well in line with Sm–Nd garnet–whole rock data, pointing to pegmatite formation at 263–280 Ma (Habler et al., 2009) and subsequent cooling below c. 500–550 °C at 240–260 Ma. Consequently, the presented data are explained by a variable net loss of Sr during the meta-pegmatite evolution in the Cretaceous.

## 6 Conclusions

Muscovite porphyroclasts from Permian metapegmatites of the Upper-Austroalpine Matsch Unit in Southern Tyrol (Italy) provide information on the behaviour of Sr in muscovite and the associated effects on the Rb–Sr geochronometer during upper greenschist facies deformation:

1. New Rb–Sr data indicate significant effects of deformation-related net loss of Sr on the Rb–Sr geochronometer. Kink planes, subgrain boundaries and cracks show major element compositional alteration and may have provided short-circuit diffusion (fast-diffusion) pathways for incompatible Sr.
2. Based on BSE images, quantitative element mapping by EMPA and single spot analyses, 3 distinct muscovite groups were identified in Permian metapegmatite: (i) Wm I represents primary magmatic Permian muscovite with almost pure muscovite endmember composition; (ii) Wm II forms portions of Ms clasts, which were affected by alteration/dissolution–reprecipitation; and (iii) Wm III appears as fine-grained microstructurally distinct Ms generation, which is largely compositionally similar to Wm II, but results from (neo)crystallisation in the rock matrix and strain shadows, or from dynamic recrystallisation in highly strained portions of coarse grained clasts (Fig. 2, Fig. 3).
3. The presented model assumes a primary magmatic muscovite population with a homogeneous ^87^Rb/^86^Sr ratio and variable net loss of Sr. The predicted present-day scattered distribution of the muscovite populations almost matches the measured distribution and confirms the role of net loss of Sr during the metapegmatite evolution in the Cretaceous.
4. All samples show a positive internal correlation of ^87^Rb/^86^Sr and ^87^Sr/^86^Sr ratios of their muscovite irrespective of their finite strain magnitude.
5. All samples, except M1201 and M1206, comprise a primary magmatic muscovite fraction that has not been significantly affected by a net loss of Sr and hence preserves the original Permo-Triassic formation/cooling age.
6. A whole-rock Rb–Sr apparent age from Permian pegmatites is in line with a pegmatite formation during the Permo-Triassic HT/LP event, suggesting that the whole rock systems have remained largely closed systems during Cretaceous tectonometamorphic overprinting at least with respect to Rb and Sr.
7. Investigation of different grain size- and magnetic-fractions from single samples provides a comprehensive dataset documenting significant systematic variations in the Rb–Sr system even at hand specimen scale. Mean Rb–Sr muscovite–whole rock apparent ages of different muscovite grain-size and magnetic fractions decrease from the weakly deformed sample M1217 (236 Ma) to the mylonitic sample M1201 (177 Ma), hence documenting a significant effect of deformation on the muscovite Rb–Sr geochronometer at upper-greenschist facies conditions.
